# Telephone-delivered psychosocial interventions targeting key health priorities in adults with a psychotic disorder: systematic review

**DOI:** 10.1017/S0033291718001125

**Published:** 2018-05-25

**Authors:** Amanda L. Baker, Alyna Turner, Alison Beck, Katherine Berry, Gillian Haddock, Peter J. Kelly, Sandra Bucci

**Affiliations:** 1 School of Medicine and Public Health, University of Newcastle, Newcastle, Australia; 2 Division of Psychology and Mental Health, School of Health Sciences, Faculty of Biology, Medicine and Health, University of Manchester, Manchester Academic Health Science Centre; Greater Manchester Mental Health NHS Foundation Trust, Manchester, UK; 3 Illawarra Institute for Mental Health, School of Psychology and the Illawarra Health and Medical Research Institute, University of Wollongong, Wollongong, Australia

**Keywords:** Cardiovascular risk, medication compliance, psychosocial telephone intervention, psychotic disorder, relapse

## Abstract

**Background:**

The mental and physical health of individuals with a psychotic illness are typically poor. Access to psychosocial interventions is important but currently limited. Telephone-delivered interventions may assist. In the current systematic review, we aim to summarise and critically analyse evidence for telephone-delivered psychosocial interventions targeting key health priorities in adults with a psychotic disorder, including (i) relapse, (ii) adherence to psychiatric medication and/or (iii) modifiable cardiovascular disease risk behaviours.

**Methods:**

Ten peer-reviewed and four grey literature databases were searched for English-language studies examining psychosocial telephone-delivered interventions targeting relapse, medication adherence and/or health behaviours in adults with a psychotic disorder. Study heterogeneity precluded meta-analyses.

**Results:**

Twenty trials [13 randomised controlled trials (RCTs)] were included, involving 2473 participants (relapse prevention = 867; medication adherence = 1273; and health behaviour = 333). Five of eight RCTs targeting relapse prevention and one of three targeting medication adherence reported at least 50% of outcomes in favour of the telephone-delivered intervention. The two health-behaviour RCTs found comparable levels of improvement across treatment conditions.

**Conclusions:**

Although most interventions combined telephone and face-to-face delivery, there was evidence to support the benefit of entirely telephone-delivered interventions. Telephone interventions represent a potentially feasible and effective option for improving key health priorities among people with psychotic disorders. Further methodologically rigorous evaluations are warranted.

Cardiovascular disease (CVD), relapse and poor adherence to psychiatric medication are key health priorities for people living with a psychotic disorder. Life expectancy is 12–19 years shorter than that of the general population (Laursen, 2011), with CVD the single largest cause of death among this group (Brown *et al.*, 2000). Rates of major health risk behaviours associated with CVD (smoking, physical inactivity, alcohol use and low fruit and vegetable intake) are also elevated (Galletly *et al.*, 2012; Morgan *et al.*, 2012). Wellbeing is further compromised by high rates of relapse (Brissos *et al.*, 2011) and although medication can reduce relapse (Alvarez-Jimenez *et al.*, 2012) rates of non-compliance are as high as 50% (Lacro *et al.*, 2002) and early discontinuation is common (Lieberman *et al.*, 2005).

Importantly, increasing evidence supports the role of psychological interventions (e.g. cognitive behaviour therapy, family therapy) for improving symptoms (Wykes *et al.*, 2008; Jauhar *et al.*, 2014), reducing relapse (Bucci *et al.*, 2016; Oud *et al.*, 2016), improving medication adherence (Barkhof *et al.*, 2012) and modifying health risk behaviours (Baker *et al.*, 2009; Banham and Gilbody, 2010; Baker *et al.*, 2012). However, of those likely to benefit from psychological interventions, only 10% or less have access (Gulliver *et al.*, 2010; Haddock *et al.*, 2014; Schizophrenia Commission, 2015). Improving access to psychosocial interventions is, therefore, an important priority if we are to improve the wellbeing of individuals living with a psychotic illness. Contrary to assumptions that people with a psychotic disorder do not have access to and/ or are unwilling to engage in technology, accumulating evidence [e.g. (Firth *et al.*, 2016; Gay *et al.*, 2016)] suggests that the potential to use technology such as telephone-based intervention delivery is huge.

As far as the authors are aware, there has been only one previous systematic review of telephone-based interventions for mental health problems. However, people with a schizophrenia spectrum disorder were included in only one study (Leach and Christensen, 2006). A more recent systematic review of telepsychiatry (telephone, internet or videoconferencing) in the assessment and treatment of people with a schizophrenia spectrum disorder included six studies (Kasckow *et al.*, 2014). However, neither review included studies targeting people with bipolar disorder. Moreover, neither reviewed the evidence for multiple key health priorities in adults with a psychotic disorder (namely relapse prevention, medication adherence and health behaviours).

## Aims of the current review

Given the poor physical and mental health of people with a psychotic disorder, limited access to healthcare and the potential promise of telephone-delivered interventions, we aim to provide an overview and critical analysis of the current state of evidence for telephone-delivered psychosocial interventions for relapse prevention, medication adherence, and modifiable CVD risk behaviours among people with a psychotic disorder (schizophrenia spectrum disorder or bipolar disorder). The focus of this review will be on person-delivered interventions using the spoken word (i.e. interventions delivered entirely by text, web and/or automated systems were excluded) and one or more psychological strategies (see published protocol for further details; Beck *et al.*, 2015).

## Methods

### Protocol and registration

This systematic review is registered with PROSPERO (Registration Number CRD42015025402) and the protocol has been published (Beck *et al.*, 2015).

### Criteria for selecting studies for this review

Methods were informed by Cochrane Guidelines for systematic reviews (Higgins and Green, 2011) and are extensively detailed in the review protocol (Beck *et al.*, 2015). The population of interest was adults (⩾18 years) with a psychotic disorder (as defined by any criteria). We included studies with populations involving adults with non-psychotic disorders only if more than 50% of participants had a psychotic disorder, or if data limited to those with psychotic disorders were available. The intervention of interest was telephone support targeting: (i) relapse prevention, (ii) adherence to psychiatric medication and/or (iii) smoking and other CVD health risk behaviours [see (Beck *et al.*, 2015) for definitions]. These domains were targeted as they represent an important avenue for improving the health and wellbeing of adults with psychosis since they are common challenges that have profound implications for the individual and are amenable to change following psychological intervention. Telephone support was defined as a person delivered intervention of at least 10 min using spoken word and one or more psychological strategies (see published protocol for further details; Beck *et al.*, 2015). The telephone support could be a standalone intervention or delivered in combination with other treatment components. However, studies with multiple components were only included if the telephone was the predominant method of intervention delivery (defined as ⩾ 50% of the total number of participant contacts conducted by telephone). Interventions delivered in any setting (e.g. community, hospital, rehabilitation or residential treatment centre, etc.) were included. The telephone support could be compared with inactive (e.g. standard care, waiting list control) and/or active controls (e.g. pharmacological and/or psychological alone and/or in combination with usual care) whereby telephone was not the predominant method of intervention delivery (e.g. individual, group, internet). Studies had to provide data for at least one of the following: (a) relapse, (b) medication adherence, (c) health risk behaviours/CVD risk, (d) process variables (e.g. treatment engagement) or (e) feasibility [see (Beck *et al.*, 2015) for definitions]. Process variables are included in Supplementary File 1. Qualitative studies were the only study design excluded.

### Search methods for identification of studies

**Fig. 1.**
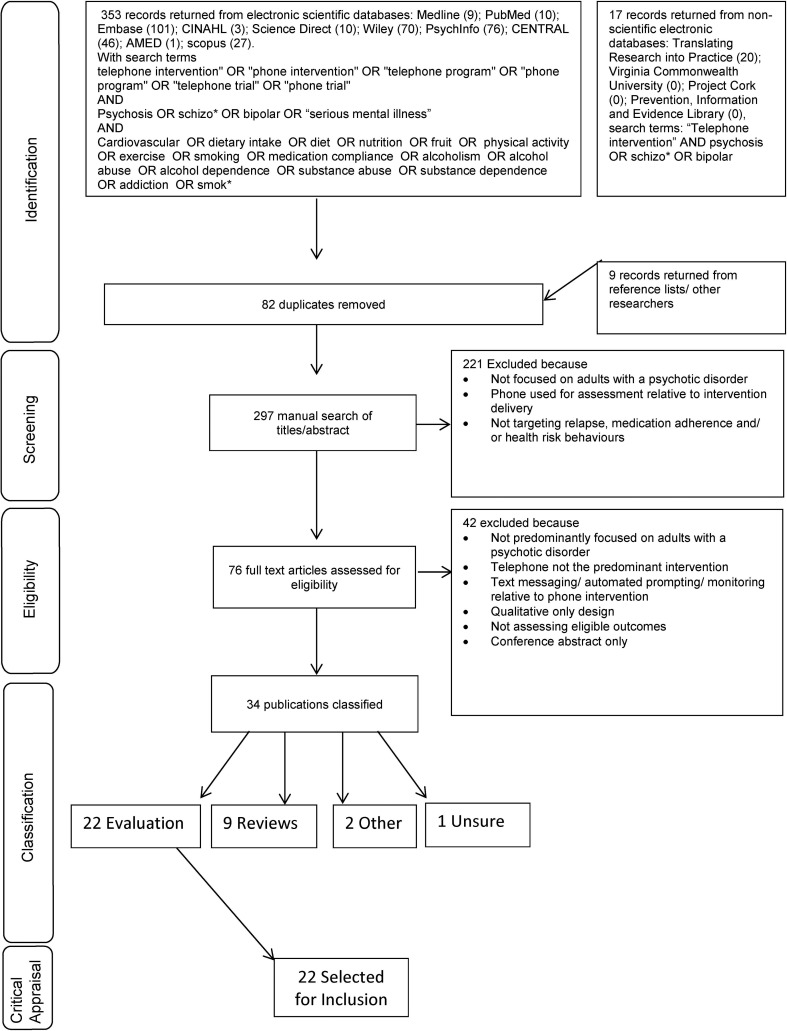
PRISMA flow diagram summarising systematic search identifying evaluations of telephone delivered psychosocial interventions for relapse prevention, medication adherence and health risk behaviours in adults with a psychotic disorder.

Figure 1 summarises the procedure used to identify studies, (see online Supplementary Appendix 1 for the full MEDLINE search strategy). Abstract, title, keywords and subject headings specific to each of the identified databases were searched. All subject headings were exploded so that narrower terms were included. No limits were placed on publication year. Publications had to be available in English. Reference lists were hand searched to identify any additional publications. Publications were organised in reference manager Endnote. The first search was run in May 2015 and re-run just before final analyses (December 2016). Articles were identified and classified according to the following steps:

#### Step 1: Identification and screening

AKB performed the searches and reviewed the titles and abstracts of the identified 297 publications and used the inclusion criteria to exclude clearly ineligible articles. If eligibility was unclear, the full-text article was accessed.

#### Step 2: Eligibility and classification

The full-text version of 76 publications was manually reviewed and 42 publications were excluded. The remaining 34 were classified as ‘evaluation’, ‘review’, ‘discussion’ or ‘other’ according to published definitions (Beck *et al.*, 2015).

#### Step 3: Cross-checking

The 76 publications from step two were cross-checked by ALB. The 22 studies independently classified as ‘evaluation’ were retained for further examination.

### Data collection and analysis

Data extraction was performed by ALB and checked by AT, SB and KB. When multiple reports of the same study were identified (Simon *et al.*, 2002, 2005, 2006) data were extracted separately and combined across data collection forms. Criteria for data extraction (detailed in the protocol; Beck *et al.*, 2015) were adapted from the Cochrane Handbook for Systematic Reviews (Higgins and Green, 2011) and the Downs and Black Scale (Downs and Black, 1998).

### Assessment of methodological quality and risk of bias

Methodological critique and assessment of risk of bias on individual studies were performed independently by ALB and AT, with final ratings made by consensus. As we included both randomised and non-randomised designs multiple tools were used.

#### Downs and Black scale

All studies were assessed against the Downs and Black Scale (Downs and Black, 1998). This scale is recommended by the Cochrane Guidelines for assessing the quality of non-randomised trials (Higgins and Green, 2011). Consistent with previous research (e.g. Baker *et al.*, 2012) two items were not used. Scoring of the final item (power) was unclear so the following convention was used: 0 = no power calculation reported; 1 = power analysis reported, but insufficient power achieved and 2 = power analysis reported and sufficient power achieved. All other items were scored per published guidelines (Downs and Black, 1998) for a total maximum of 27, with higher scores reflecting greater methodological quality.

#### PEDro scale

Randomised controlled trials (RCTs) were assessed against the 11 item Physiotherapy Evidence Database (PEDro) scale (Maher *et al.*, 2003), a widely implemented and validated tool for assessing the quality of randomised trials. As per above, the two items regarding blinding were not used (e.g. Spring *et al.*, 2011, Baker *et al.*, 2012). The remaining nine criteria were assigned a yes (1 point) or no (0 points) rating, and a quality score ranging from 0 to 8 points was calculated for each study.

### Cochrane collaboration's risk of bias tool

Risk of bias (within and across all studies) was assessed using the Collaboration's Risk of Bias tool, as described in the *Cochrane Handbook for Systematic Review of Interventions* (Higgins and Green, 2011). Each item was judged as being high, low or unclear risk as per the criteria provided by Higgins and Green (Higgins and Green, 2011). Given the evidence that sequence generation and allocation concealment represent particularly important potential sources of bias, studies were deemed to be at the highest risk of bias if either item was scored as ‘high’ or ‘unclear’.

## Summary measures

A study was considered to have a positive outcome if more than 50% of the reported outcome measures (primary and secondary) demonstrated a between-group difference in favour of the telephone group at the treatment end. Positive maintenance outcome(s) were identified when this effect was evident at short and/or medium and/or long-term follow-up (1–6; 7–12 and >12 months after intervention completion, respectively).

## Synthesis of results

Comparability of study design and outcome measures across studies was assessed by a consultant statistician to determine the possibility of conducting meta-analyses on RCTs to examine effects on relapse, medication adherence and smoking and other health behaviours and CVD risk. A narrative synthesis of the findings was conducted, structured around intervention type, outcome, population and methodological quality. As Clinical Guidelines recommend an improved focus on personally meaningful recovery (e.g. quality of life, functioning) relative to traditional clinical outcomes (e.g. symptoms and relapse) in mental health care, to help inform clinical practice, the assessment, reporting and/ or change in these additional outcomes is also central to the structure of the review.

## Results

### Participant Characteristics

Across all studies, the total number of participants was 2473, with 867 in relapse prevention, 1273 in medication adherence and 333 in smoking and/or other health risk behaviour studies (see online Supplementary Table S1). The average age was 40.7 years (41.9 in relapse prevention, 39.5 in medication adherence and 42.2 in smoking and/or other CVD risk behaviours). Overall, the percentage of males across the studies was 50.1%. However, there was a higher percentage of males in studies of schizophrenia samples (64.5%) compared with studies of bipolar (37.7%) and mixed samples (44.2%). No study used a first episode sample.

### Study characteristics

The 22 papers comprised a total of 20 trials, with Simon *et al.* (Simon *et al.* 2002, 2005, 2006) reporting on the same study. There were 16 controlled (Table 1) and four single-arm (Table 2) studies. Nine trials recruited people with bipolar disorder, six with schizophrenia spectrum disorder, four with schizophrenia and one a range of diagnoses (see online Supplementary Table S1). For the RCTs the telephone was the sole method of intervention delivery in one relapse prevention (Beebe, 2001) and three medication adherence trials (Salzer *et al.*, 2004; Cook *et al.*, 2008; Beebe *et al.*, 2016). For the studies without a comparison condition, the intervention was delivered entirely by telephone for two relapse prevention (Miklowitz *et al.*, 2012; Boardman *et al.*, 2014) and one healthy lifestyle (Baker *et al.*, 2014) study.

**Table 1.** Summary of findings as a function of study focus (relapse prevention *v*. medication adherence *v*. smoking/healthy lifestyles) and comparison condition (active *v*. treatment as usual), structured in descending order according to the quality rating

Author study design	Clinical group	*N*	Intervention delivery methods				Outcomes			Quality rating			
			Telephone condition		Comparison condition								
			Phone	Face to Face^f^	Phone	Face to Face^f^	Primary	Outcome in Favour of	Secondary/Process	Outcome in Favour of	Downs & Black	PEDRO	Overall Risk of Bias
**Relapse prevention**													
***Telephone v. active comparison condition***													
Komatsu *et al*. (2013) RCT	SZ	Phone (*n* = 22) *v.* control (*n* = 23)	✓ Weekly (? min) intervention × 12 months	✓ Home visits (as indicated) to support intervention compliance	✓ Weekly (? min) assessment ×12 months	⨯	Number of hospitalisations two treatment (9.1%) *v*. 8 control (34.8%), *p* = 0.071 Period until hospitalisation: Longer for treatment than control, log rank, 4.53, *p* = 0.0033 Risk of Rehospitalisation Reduced in treatment *v*. control (hazard ratio = 0.21, 95% CI 0.04–0.99, *p* = 0.049; Number needed to treat = 4; 95% CI = 2.1–35.5) Total number of rehospitalisation days: 37 intervention *v*. 710 control, *p* = 0.023. Number of inpatient days on each hospitalisation Lower for treatment (18.5 days) than control (88.8 days); *p* = 0.036	T T T T T	Non-hospitalised relapses (due to worsening psychiatric symptoms, based on physician judgement). No significant difference between groups (*p* value not reported) Psychiatric symptoms (BPRS) at the time of rehospitalisation No significant difference between groups for mean change in total scores (*p* = 0.135) Posthoc: Mean change in total BPRS scores at relapse was less for treatment, changing by 11.3 points compared with 17.2 for control (*p* = 0.019)	NSD NSD T	21 – –	6 – –	Low – –
Castle *et al*. (2007) Pilot RCT	BP	Phone (*n* = 8) *v*. Control (*n* = 9)	✓ Weekly (? min) follow-up phone checks× 12 weeks	✓ Weekly 90 min group × 12 + Monthly 90 min booster group × 3	✓ Weekly (? min) supportive phone calls × 12 weeks	⨯	Rates of relapse (meet DSM-IV criteria for manic or depressive episode, and/or required hospital admission) Treatment = 1 *v*. control = 4 relapsed over the 10-month period (*p* = 0.3).	T	General functioning (GAF): Significant improvement for Treatment: baseline M = 56.0 (s.d. = 12.0), 6m M = 72.0 (s.d. = 11.0) *v*. Control baseline M = 61.0 (s.d. = 10.0), 6m M = 62.0 (s.d. = 9.0); *p* < 0.05. Quality of life (WHOQoL BREF): Social relationships Significant improvement for Treatment: baseline M = 52.0 (s.d. = 28.0), 6m M = 60.0 (s.d. = 14.0) *v*. Control baseline M = 68.0 (s.d. = 19.0), 6m = 66.0 (s.d. = 21.0), *p*<0.05). Satisfaction with health/Physical Health/Psychological Health/Environment No significant change within or between groups reported Depression (MADRS) No significant change within or between groups reported Mania (YMRS) No significant change within or between groups reported Medication adherence (MARS) No significant change within or between groups reported	T T NSD NSD NSD NSD	18 – – – – –	8 – – – – –	High – – – – –
Wenze *et al.*(2015) Pilot RCT	BP	Phone (*n* = 14) *v*. Control (*n* = 16)	✓ 11× (15–30 min) weekly for 1 month then at decreasing frequency for 4 months	✓ 3× individual 1× family across 1month	⨯	✓ 3× Ax's (Baseline, 3 and 6 months) & treatment providers given a written summary	Faster and greater improvement for treatment *v*. control (all *p* < 0.05) for:		No secondary outcomes specified. Process Variable:	**–**	18	6	High
							Depression (QIDS-C; ƒ^2^ = 0.24)	T	Attendance:	?	–	–	–
							Mania (CARS-M; ƒ^2^ = 0.37)	T	M = 2.71 (s.d. 0.73) in-person individual		–	–	–
							Valued living (VLQ; ƒ^2^ = 0.31)	T	M = 0.36 (s.d. 0.50) in-person family	–	–	–	–
							Functional impairment (WHODAS 2.0 Brief; ƒ^2^ = 0.12)	T	M = 9.50 (s.d. 4.67) individual phone Significant Others completed M = 4.07 (s.d. 4.58) phone	– –	– –	– –	– –
							‘Marginal effect in favour of treatment’ (*p* < 0.10) for:			–	–	–	–
							Suicidal ideation (QIDS-C item 12^a^)	NSD		–	–	–	–
							Medication adherence (MCQ^a^)	NSD		–	–	–	–
							Mental Health Care Service Use – including hospitalisation (THxI^a^)	NSD		–	–	–	–
							Days using drugs (excl. alcohol; TLFB^a^)	NSD		–	–	–	–
							Satisfaction (CSQ-8): Treatment significantly higher (29.67; s.d. = 2.45 *v.* 25.17; s.d. = 4.61, *p* = 0.02)	T		–	–	–	–
							Expectancies for Improvement (CES) High across both groups. ‘Marginally higher’ for treatment Baseline M = 40.07 (s.d. = 8.96) *v*. control M = 34.13(s.d. = 8.85) (out of a possible total of 54); *t*_(27)_ = −1.80, *p* = 0.08). 6m M = 29.67(s.d. = 2.45) *v.* control M = 5.17(s.d. = 4.61) (out of a possible total of 32); *t*_(19)_ = −2.65, *p* = 0.02	T		–	–	–	–
Castle *et al*. (2010) RCT	BP	Phone (*n* = 32) *v.* control (*n* = 40)	✓ Weekly (? min) follow-up phone checks× 12 weeks	✓ Weekly 90 min group× 12 + Monthly 90 min booster group×3	✓ Weekly (? min) supportive phone calls×12 weeks	⨯	Survivor function for first relapse (of any type, meeting DSM-IV-TR criteria): Lower rates of relapse for treatment (hazard ratio = 0.43, 95% CI 0.20–0.95; *p* = 0.04).	T	Depression (MADRS) No significant change within or between groups (*p* = 0.8)	NSD	17	7	Low
							No relapse *v*. at least one relapse (DSM-IV-TR criteria): No relapse (23 treatment *v.* 18 control) *v.*one or more relapse (nine treatment *v.* 22 control) *p* = 0.03.	T	Mania (YMRS) No significant change within or between groups (*p* = 0.3)	NSD	–	–	–
							Fraction of time spent unwell (during the 9-month follow-up): Significantly less time unwell for treatment (*p* = 0.02)	T					
Beebe (2001) Pilot Randomised Post-test Control Group	SZ	Phone (*n* = 15) *v*. control (*n* = 22)	✓ Weekly intervention (~10 min)×3 months	⨯	✓ 2 × assessment (1–3 min) at 6 and 12 months	⨯	Community survival (number of days until first psychiatric rehospitalisation or study end) Phone M = 81.4 *v*. control 78.3 (*p* = 0.70)	NSD	No Secondary Outcome(s) Specified	**–**	14	3	High
							Length of rehospitalisation/s (days): Phone M = 19.0 *v.* Control 26.1 (*p* = 0.51)	NSD		–	–	–	–
							Frequency of rehospitalisation: Phone 13% *v*. control 23% readmitted (*p* = 0.52)	NSD		–	–	–	–
***Telephone vs. treatment as usual***													
Simon et al (2002, 2005, 2006) RCT	BP	Phone (*n* = 212) *v.* Control (*n* = 229)	✓ Monthly (? Duration) over 24 months	✓ 1× individual assessment/care planning Up to 48 group sessions (5× weekly, then twice-monthly) Outreach visits ‘as needed’	⨯	⨯	Mania severity (PSR): Lower in treatment group throughout the 2-year follow-up (*p* < 0.04). If symptomatic at baseline treatment had a significant effect on mean mania scores (*z* = 2.27, *p* < 0.02).	T	Mania duration (weeks PSR scores >3) Significantly lower for treatment (M = 19.2, s.d. = 20.2) *v*. control (M = 24.7, s.d. = 24.3), *p* = 0.01.	T	25	8	Low
							Depression severity (PSR) No significant difference between the two groups (*z* = 0.19, *p* = 0.85)	NSD	Depression duration (weeks PSR scores >3) No significant treatment effect on time (47.6 *v.* 50.7 weeks; *F*_1_ = 0.56, *p* = 0.45).	NSD	–	–	–
									Outpatient Mental Health, Appts (Computerised Registration Data)	NSD	–	–	–
									Medication Management (*p* = 0.5)	–—	–	–	–
									Individual Psychotherapy (*p* = 0.45)	–	–	–	–
									Psychiatric Hospitalisation (*N*) *p* = 0.91	NSD	–	–	–
									Medication Use	NSD	–	–	–
									Mood Stabiliser: *p* = 0.59	–	–	–	–-
									Antidepressant: *p* = 0.85	–	–	–	–
									Antypical Antipsychotic: *p* = 0.23	–	–	–	–
									Cost	NSD	–	–	–
									Outpatient mental health visit: *p* = 0.53	–	–	–	–
									Psychiatric hospitalisation: Treatment *p* = 0.34	–	–	–	–
									Psychotropic drug prescription *p* = 0.08	–	–	–	–
									Total Cost Intervention M = $8046 (s.d. = 5974) *v.* control M = $6743(6695), *p* = 0.06	–	–	–	–
									Process Variable:				
									Attendance:	?	–	–	–
									203 (95.8%) completed >1 telephone & 180 (84.9%) completed >12 telephone contacts. 137 (64.6%) attended > 1 group session, 125 (59.0%) completed 5 weekly sessions & 108 (50.9%) continued for >12 months	–	–	–	–
Javadpour et al. (2013) RCT	BP	Phone (*n* = 54) *v.* control (*n* = 54)	✓ 18× monthly (~10 min) follow-up care for 18 months	✓ 8×weekly (~50 min) individual sessions	⨯	⨯	Number of hospital admissions (hospital file audit): M = 0.22 treatment *v*. M = 1.41 control hospitalised due to bipolar disorder, *p* = 0.000.^b^	T	No secondary outcomes specified	–	16	6	High
							Depression severity (HDRS): Lower for treatment (baseline M = 4.2; 6m M = 6.3; 12m M = 6.0; 18m M = 5.8) *v.* control (baseline M = 5.2; 6m M = 10.2; 12m M = 11.2; 18m M = 11.2), *p* = 0.000^b^	T	Process Variable:				
							Mania (BRMAS): Lower for treatment (baseline M = 4.2; 6m M = 4.6; 12m M = 4.9; 18m M = 4.1) *v*. control (baseline M = 4.3; 6m M = 8.8; 12m M = 10.0; 18m M = 7.3), *p* = 0.000.^b^	T	Attendance: Mean number of face-to-face sessions attended was 7.3 & 15.3 of the telephone follow-up programme	?	–	–	–
							Depression or mania recurrence (HDRS >7 or BRMAS >9): Fewer recurrences for treatment (M = 0.77) *v.* control (M = 2.02) *p* = 0.000)^b^	T					
							Medication adherence (MARS): Higher for treatment (6m M = 7.9; 12m M = 7.8; 18m M = 7.9) *v.* control (6m M = 4.7; 12m M = 4.0; 18m M = 3.7), *p* = 0.008.^b^	T					
							Quality of life (WHOQOL-BREF) All subscales higher for treatment *v*. control (all *p* = 0.000).^b^ Physical health M = 63.8 *v.* M = 53.3; Mental Health M = 66.7 *v.* M = 54.3; Social Health M = 74.1 *v.* M = 51.7; Environment M = 65.1 *v.* M 48.9	T					
Price (2007) Pilot RCT	SZ/ SZ-A	Phone (*n* = 7) *v*. control (*n* = 6)	✓ 1–2 calls within 2 wk of discharge +190 phone minutes to use over three months	✓ 1× structured interview 2–3 days before discharge	⨯	✓ Structured Interview 2–3 days before discharge	Number of hospital readmission days (through hospital records): Treatment M = 4.0 (range 0–19) *v.* 10.33 (range 0–28) control (*p* = 0.2389)	NSD	No secondary outcomes specified	–	13	6	High
							Compliance with outpatient appointments (reported by case managers): Treatment kept appointments 5/7 times (71.4%) *v.* 3/6 (50%) controls, (*p* = 0.4126)	NSD	Process Variable: Attendance: Two participants used all 190 pre-paid mobile minutes in the first week after discharge, so that 30-min increments were then added (amount unspecified)	?	–	–	–
							Compliance with medications (reported by case managers and participants): 4/7 (57%) intervention *v*. 2/6 (33.3%) control (*p* = 0.3834)	NSD					
Haddock *et al.* (2017) Partially randomised patient preference trial	SZ Spectrum (incl. BP)	Phone (TS) (*n* = 35) Group + Phone (HS) (*n* = 33) Control (*n* = 33)	✓ TS: Weekly (60 min)×9 months HS: As per TS	✓ TS: Up to 2 × individual (initial session & final session ‘if desired’) HS: As per TS + up to 12 bi-weekly, 2-hour group sessions delivered over 6 months	⨯	✓ Trial Ax's (Baseline, 9 & 15 months)	Ratings on strengths of choices: No significant differences in strength of preference between groups, with strong preferences reported (>8/10).	NSD	Symptoms: No within or between group differences for positive and negative symptom severity (PANSS)^c^	NSD	16 –	– –	High –
							Process of Recovery (QPR) No overall treatment effect at 9 (*p* = 0.58) or 15 months (*p* = 0.82)	NSD	Distress in relation to positive symptoms (PSYRATS)^c^ Personal and social functioning (PSP)^c^	NSD NSD	– –	– –	– –
							Recovery from Psychosis (SEPS) Negative Impacts Subscale 15-month follow-up, significant difference in adjusted means in favour of the control group (16.85 units; 95% CI 1.36–32.35, *p* = 0.03)	C	Depression (CDS)^c^ Anxiety (BAI)^c^ Therapeutic alliance (WAI). Comparable to prior trials of face-to-face CBT for psychosis. Strength of preference for chosen treatment arm significantly associated with higher client rated therapeutic alliance [*r* (22) = 0.49, *p* = 0.021]	NSD NSD NSD	– – –	– – –	– – –
									Process Variable				
									Attendance: *Telephone sessions*: TS: 15.8 (s.d. 10.8); HS: 9.8 (s.d. 9.5); *Group sessions*: 3.0 (s.d. 3.6). *Total telephone DNAs*: TS: 4.4 (s.d. 5.0); HS: 4.5 (s.d. 4.9). *Duration (mins)* TS: 40.6 (s.d. 9.2); HS 39.6 (s.d. 1.3). TS completed significantly more telephone sessions (*p* < 0.05).	?	–	–	–
									Satisfaction: Preferred treatment associated with satisfaction. (Dis)satisfaction with current treatment also informed treatment preference	?	–	–	–
**Medication adherence**													
***Telephone v. active comparison condition***													
No studies													
***Telephone v. treatment as usual***													
Montes *et al*. (2010) RCT	SZ	Phone (*n* = 456) *v*. control (*n* = 472)	✓ 3×monthly (? min)	✓ Psychiatrist visit (as indicated)	⨯	✓ 1×psychiatrist visit at 4 months	Clinician rated adherence to antipsychotic medication (RAT): 96.7% treatment *v*. 91.2% control, *p* = 0.0007. (Increase in % adherence: 8.5% treatment *v.* 1.1% control).	T	Illness severity (CGI-SCH-SI): Positive Symptoms: Treatment (M = 2.4, 95% CI = 2.28–2.44) superior to control (M = 2.5, 95% CI = 2.40-2.55), *p* = 0.02^b^ Negative Symptoms	T NSD	22 – –	7 – –	Low – –
							Treatment significantly more likely to be adherent than control (adjusted OR = 3.3, 95% CI = 1.6–6, *p* = 0.0001)		Depressive Symptoms Cognitive Symptoms Global Symptoms:	NSD NSD NSD	– – –	– – –	–– –
							Greater % of non-adherent patients becoming adherent in treatment (10.4%, *p* = 0.0013) *v.* control (5.2%, *p* = 0.43)		Clinical improvement (CGI-SCH-DC): Positive Symptoms Treatment (M = 3.0) superior to control (M = 3.2), *p* = 0.0088^b^	T	–	–	–
							Significantly higher percentage of treatment (25.7%, *n* = 109) improved adherence at the end of the study vs. control (16.8%, *n* = 74), *p* = 0.0013		Depressive Symptoms Treatment (M = 3.0) superior to control (M = 3.2), *p* = 0.01^b^ Cognitive Symptoms	T T	– –	– –	– –
							Hospitalisations (RAT): eight treatment (1.8%) *v.* 5 control (1.1%), ns^c^	NSD	Treatment (M = 3.2) superior to control (M = 3.4), *p* = 0.02^b^ Global Symptoms	T	–	–	–
									Treatment (M = 3.1) superior to control M = 3.3), *p* = 0.0099^b^		–	–	–
									Attitudes to medication (DAI-10): Treatment (M = 6.1, 95% CI = 5.75–6.35) superior to control (M = 5.2, 95% CI = 4.91–5.48), *p* < 0.0001^b^	T	–	–	–
									Quality of Life (EQ-5D): 0.80 treatment *v*. 0.78 control, (*p* = 0.07).	NSD	–	–	–
									Process Variable:				
									Attendance: 865 attended at least one follow-up phone call. Of 34 patients classified as non-adherent during at least one telephone call, 20 kept their psychiatrist appointment	?	–	–	–
Beebe *et al*. (2016) RCT	SZ/SZ-A	Phone (*n* =?) *v*. control (*n* =?) (Total *N* = 140)	✓ Weekly (? min) over 3 months	⨯	⨯	⨯	Self-reported medication adherence (MARS): ns^c^.	NSD	No secondary outcomes specified	–	13	2	High
							Medication adherence self-efficacy (MASES): ns^c^.	NSD					
							Symptom level (PANSS): ns^c^	NSD					
Salzer *et al.* (2004) RCT	SZ Spectrum	Phone (*n* = 13) *v*. control (*n* = 10)	✓ Weekly (<10 min/session) over 52 weeks	⨯	⨯	⨯	‘Treatment effect sizes in the direction of telephone’ for: Distress from side-effects ES = 0.85 (*p* = 0.06) Number of extreme side effects ES = 0.85 (*p* = 0.04) Insight ES = 0.64 (*p* = 0.14);	T T NSD	No secondary outcomes specified Process Variable Attendance: 15/18 intervention (84%) >1 session, 14/15 at least 35/52 scheduled weeks. M = 2.01 calls needed to make one contact. Average contact length: 8.54 minutes (range 5.31–12.17). Average total phone time: 4 h 32 min (range 35 min– 8 h 19 min)	– ?	9 –	2 –	High –
							Attitudes toward medication ES = 0.58 (*p* = 0.11)	NSD				–	–
							Number of side-effects ES = 0.60 (*p* = 0.16);	NSD					
							Symptoms and functioning ES = 0.26 (*p* = 0.54);	NSD					
							Staff relationships ES = 0.64 (*p* = 0.12);	NSD					
							Treatment satisfaction ES = 0.50 (*p* = 0.24).	NSD					
							No significant differences for:						
							Subjective response to medication ES = 0.01, *p* = 0.96	NSD					
							Self-reported treatment adherence: ES = .02, *p* = 0.87	NSD					
Cook *et al.* (2008) Non-randomised controlled trial, pre-post.	Range of diagnoses (including BP and SZ-A)	Phone (*n* = 51) *v*. control (*n* = 151).	✓ 1 x initial screening (? min) ‘Low-risk’ = toll free number + 1× follow-up at 6 months ‘At-risk’ = mean 3.5 (~11 min) completed calls over an average of 4.4 months	⨯	⨯	⨯	Emergency Department (ED) utilisation (healthcare plan administrative data). M = 1.11 treatment *v.* M = 5.03 control; *p*<.001; ES 0.28^b^	T	Pharmacy-based adherence (< = 14-day gap between prescription fill dates). 6m adherence: 48% treatment *v*. 26% control, *p* = 0.004, ES = 0.20.	T	19	–	High
							Exploratory PostHoc: Treatment group: 1.5 times pre-intervention *v*. 0.39 times during the intervention (*p* < 0.001).		Self-reported adherence (Adherent =>80% doses in last 7 days): 6m adherence = 50% treatment ‘which was higher than the comparison rate’ (not specified) *p* = 0.002, ES = 0.22).	T	–	–	–
									Process Variable:				
									Attendance: Nurses made a mean of 2.1 attempts per completed call. Participants at risk for non-adherence (90%): An average of 7.2 call attempts; 3.5 calls over 4.4 months; 11 min/call; 38 min’ total contact/participant	?	–	–	–
**smoking/healthy lifestyles**													
***Telephone v. active comparison condition***													
Baker *et al*. (2015) RCT^d^	SZ Spectrum (Incl. BP)	Phone (*n* = 113) *v.* HL (*n* = 112)	7× weekly, 3× f/nightly, 6×monthly (~10 min/session) over 24 weeks	1× initial (90 min) Weeks 4 and 8 (30 min each)	⨯	1× initial (90 min) 7× weekly, 3× f/nightly, 6×monthly (~60 min/session) over 24 weeks	10-year CVD risk (ASSIGN score):		Psychiatric symptomatology (BPRS)		22	7	Low
							15w: Improvement in both groups. No significant between group difference (*p* = 0.420)	NSD	15w: Stable in both groups. No significant between group difference (*p* = 0.632)	NSD	–	–	–
							12m: Improvement only in telephone: Phone M = −1.6, 99% CI = −3.2 to −0.0; *p* = 0.009; HL = −0.7, 99% CI = −2.4,1.0, *p* = 0.276). No significant between group difference (*p* = 0.789)	?T	12m: Improvement only in telephone: Phone = −4.6, 99% CI = −8.8, −0.4; *p* = 0.005; HL = −0.5, 99% CI = −4.5, 3.5, *p* = 0.758). No significant between group difference (*p* = 0.032)	?T	–	–	–
							Smoking 7-day point prevalence (biochemically validated by a CO reading ⩽ 10 ppm):		Psychiatric symptomatology (BDI-II) 15w: Stable in both groups. No significant between group difference (*p* = 0.915)	NSD	–	–	–
							15w: improvement in both groups. No significant between group difference (*p* = 0.233)	NSD	12m: Improvement only in HL: Phone = −3.8, 99% CI = −8.1, 0.4; *p* = 0.018; HL = −3.6, 99% CI = −6.8, −0.3, *p* = 0.005). No significant between group differences (*p* = 0.940)	C?	–	–	–
							12m: Improvement only in telephone (expired CO): Phone M = −7.9 (99% CI = −13.6, −2.1), *p*<.001 *v*. HL M = −4.9 (99% CI = −10.7 to 0.8), *p* = 0.026. No significant between group difference (*p* = 0.864)	?T	Global functioning (GAF): Improvement in both groups 15w & 12m. No significant between group differences at either time point	NSD	–	–	–
							Smoking reduction status (at least 50% relative to baseline): Significant reduction in both conditions at 15w and 12m. No significant between group difference at either time point (*p*’s>0.099).	NSD	Weight and its impact on quality of life: (IWOQOL-Lite): Stable in both groups at 15w and 12m. No significant between group difference at either time point (*p*’s>0.556)	NSD	–	–	–
							Cigarettes per day: Significant reduction in both conditions at 15w and 12m. No significant between group difference at either time point (*p*’s>0.565).	NSD	Quality of Life (SF-12): Stable in both groups at 15w and 12m. No significant between group difference at either time point (*p*’s >0.042).	NSD	–	–	–
									Health Behaviours & Biomedical Measures Stable in both groups at 15w and 12m. No significant between group differences	NSD	–	–	–
									Treatment retention: Significant overall difference between phone (mean 12.4, s.d. 5.2) & HL (mean 9.2, s.d. 6.0). 67% (76/113) of phone had high levels of attendance (9–17 sessions) *v*. 48% (58/122) of HL condition (*p* < 0.001).	T	–	–	–
									Smoking cessation for those attending more (9–17) *v*. fewer (1–8) sessions: 7-day point prevalence abstinence 15w 16.0% more session *v*. 4.0% fewer session (*p* = 0.006) 12m (*p* = 0.199)	?T	–	–	–
									Reduction of 50% or greater CPD 15w: 51% more session *v.* 16% fewer session (*p* < 0.001) 12m: 25% more session *v.* 6.9% fewer session (*p* < 0.001)	?T	–	–	–
Kilbourne et al. (2012) Pilot RCT	BP	Phone (*n* = 34) *v.* Control (*n* = 34)	Monthly (20 min/session) delivered across the remaining 5 months of the intervention period	4× weekly group sessions (2 h/session)	⨯	⨯	Cardiometabolic risk (BMI & Blood Pressure) Stable in both groups. No between group differences (all *p*’s >0.58) Health related QoL (SF-12): Mental and Physical Component Subscales Stable in both groups. No between group differences (all *p*’s >0.38)	NSD NSD	*Post-hoc* exploratory analyses for participants with elevated cardiometabolic risk (BMI⩾30 or systolic BP>140): Greater improvement in intervention for functioning and depressive symptoms (*p* = 0.04 for both) Not significant after correcting for multiple comparisons	T	21	7	High
							Functioning (WHODAS): ‘trend in favour of intervention’: ES 0.20 (*p* = 0.11).	?T	12 Month Service Utilisation No significant differences between groups. Process Variable:	NSD	–	–	–
							Symptoms (Internal State Scale):		Attendance: 79% of intervention participants completed at least three self-management sessions (i.e. >80% of session topics). The mean number of monthly follow-up contacts by phone or in-person was 4.5 (s.d. 1.5)	?	–	–	–
							Depressive: ‘trend in favour of intervention’: ES 0.23 (*p* = 0.15) Manic: Stable in both groups. No significant differences between groups (*p* = 0.68)	?T NSD					
Heffner *et al*. (2015) Pilot Two sequential single arm studies:^e^	BP	Phone (*n* = 6) *v.* In-person (*n* = 10)	10× 30 min sessions delivered weekly	⨯	10× 30 min sessions delivered weekly	⨯	Acceptance (AIS): Comparable change: 55% phone *v.* 54% in-person average increase from baseline	–	Depression (MADRS in person; PHQ-9 phone). ‘No clinically significant change in either group’ Mean change for phone of −1.2 (s.d. = 9.9) *v.* −1.3 (s.d. 4.0) in-person.	**–**	13	–	High
							7-day point prevalence abstinence (TLFB): End of treatment: 33% phone *v*. 40% in-person 1 m:17% phone *v*. 30% in-person	–	Mania (YMRS in person; ASRM phone): ‘No clinically significant change in either group’ Mean change for phone of 0.8 (s.d. 4.3) *v*. −0.1 (s.d. 1.1) in-person	**–**	–	–	–
							4-week prolonged abstinence (TLFB): End of treatment: 30% in-person *v*. 17% phone; 1m: 17% phone *v*.10% in-person Cigarettes/day: (50% or greater reduction between baseline and end of treatment) 67% phone *v.* 50% in-person	– –	Adherence Mean attendance was 8.3/10 sessions (s.d. 2.2) in-person; 6.7 (s.d. 2.9) for telephone. The proportion of treatment completers who used at least 80% of the NRT was 62.5% for in-person & 0% for phone. Average NRT adherence was 72.8% (s.d. 32.0) for in-person & 40.2% (s.d. 18.7) for phone condition.	**–**	–	–	–
							*Notes: CO verification in the in-person condition.* *Missing = smoking imputation for all cessation outcomes and return to baseline smoking for the reduction outcomes.*	–	Treatment satisfaction 100% phone and 90% in-person participants found the intervention helpful and would recommend the treatment. Adverse events: four psychiatric events (no suicide attempts) for phone *v*. four psychiatric (including one suicide attempt) in-person	**–**	–	–	–
***Telephone v. Treatment as Usual***													
No studies													

*Note*:

a Cohens ƒ2 not reported.

b s.d. not reported.

c *p* value not reported.

d Findings presented as mean change unless otherwise specified.

e Within subjects analysis only.

f Any face-to-face elements that are specified in addition to routine care.

ƒ2, Cohen's Effect Size; AIS, Acceptance of Illness Scale; Ax's, Assessments; BAI, Beck Anxiety Inventory; BDI, Beck Depression Inventory; BP, Bipolar; BPRS, Brief Psychiatric Rating Scale; BRMAS, Bech–Rafaelsen Mania Scale; CARS-M, Clinician Administered Rating Scale for Mania; CDS, Carroll Depression Scale; CES, Credibility and Expectancy Scale; CGI-SCH, Clinical Global Impression-Schizophrenia (-DC, degree of change; -SI, Severity of illness); CPD, Cigarettes per day; ns, non-significant; CSQ-8, Consumer Satisfaction Questionnaire; DAI-10, Drug attitude inventory; EQ-5D, EuroQol five dimensions questionnaire; ES, effect size; FTND, Fagerstrom Test for Nicotine Dependence; GAF, Global Assessment of Functioning; HDRS, Hamilton Rating Scale for Depression; IWOQOL-Lite, Impact of Weight on Quality of Life; M, Mean; MADRS, Montgomery Asberg Rating Scale; MARS, Medication Adherence Report Scale; MASES, Medication Adherence Self-Efficacy Scale; MCQ, Medication Compliance Questionnaire; NSD, No significant difference; OTI, Opiate Treatment Index; PANSS, Positive and Negative Symptoms Scale; PHQ-9, Patient health questionnaire; PSP, Personal and Social Performance Scale; PSR, Psychiatric Status Rating; PSYRATS, Psychotic Symptoms Rating Scales; QPR, Questionnaire about the Process of Recovery; RAT, Register of Adherence to Treatment; s.d., Standard Deviation; SEPS, Subjective Experience of Psychotic Symptoms; SERS, Self Esteem Rating Scale; SF-12, Short Form Health Survey; SZ, Schizophrenia; SZ-A, Schizoaffective; THxI, Treatment History Interview; TLFB, Timeline Follow Back; VLQ, Valued Living Questionnaire; WAI, Working Alliance Inventory; WHODAS, WHO Disability Assessment Schedule; WHOQOL-BREF, WHO Quality of Life Brief Scale; YMRS, Young Mania Rating Scale.

**Table 2.** Key outcomes for studies without a comparison condition (structured in descending order according to quality rating)

Author study design	Clinical group	*N*	Intervention delivery methods		Primary outcome	Summary of effect	Secondary outcome/ process variable	Summary of effect	Quality rating	
			Telephone	Face to face					Downs and black	Overall risk of bias
**Relapse prevention**										
Miklowitz *et al.* (2012) Two sequential single arm studies	BP	19	✓ 5–6× weekly; bi-weekly and then monthly (30 min) for the 4–5-month duration of the intervention + Daily texts and emails	⨯	Depression (QIDS-SR): Baseline previous 4 week mean 7.4 (s.d. 5.6). Reduction of −0.11 points per week over time Mania (ASRM): Baseline previous 4-week median score was 1. Average increase of 0.08 points per week over time Changes in self-reported knowledge of mood management strategies (BMMQ): Significant increase in total knowledge score, mean of 54.2 (s.d. 8.8) at week 1–66.9 (s.d. 9.5), *p* < 0.001 Treatment retention: 19 agreed, 17 completed all sessions in the required time frame (mean ± s.d.: Pilot I: 9.2 ± 3.4, range: 5–17 weeks and Pilot II: 7.6 ± 0.9, range: 7–9 weeks). 94.7% follow-up rate Facilitators’ fidelity to the manual (TCAS): 78.2% of rated sessions met the threshold for ‘good’ fidelity (>5)	? (Data did not meet normality assumptions) ? (Data did not meet normality assumptions) Improvement Feasible Good fidelity	No secondary outcomes specified – Process Variable: Attendance: Mean time to complete program of 9.2 weeks (s.d. 3.4; range 5–17) for Pilot I & 7.6 weeks (s.d. 0.9; range 7–9) for Pilot II. Participants responded to an average of 81% of the daily text or email prompts during treatment. Number of telephone sessions delivered was not reported –	– – ? – –	16 – – – –	High – – – –
**Medication adherence**										
McKenzie & Chang (2015) Single group pre-post	BP	14	✓ 2× (20–30 min) delivered weekly	✓ 1× initial (45–60 min) session	Self-reported adherence MARS: Improved adherence: mean 4.3 (s.d. 2.6) to 2.2 (s.d. 1.6) (*p* < 0.01) Total rate of adherence (TLFB): 67.8–94.3% (*p* < 0.01)	Improvement Improvement	Motivation to change (10-point Likert scales): Significant improvement on importance, motivation and confidence regarding medication adherence Pre-test M = 24.7 (s.d. 5.4) to mid-test M = 27.1 (s.d. 2.7) (*p* = 0.025); Mid-test to post-test M = 28.7 (s.d. 1.7) (*p* = .001); Pre- to post-test (*p* = 0.004) Self-efficacy (SEAMS): Significant improvement, Pre-test M = 26.8 (s.d. 5.9) to mid-test M = 29.6 (s.d. 3.6) (*p* = 0.026), Mid-test to post-test M = 33.4 (s.d. 3.5) (*p* = 0.000) Pre- to post-test (*p* = 0.001) Treatment satisfaction (5 × 5-point Likert scales). Mean scores for each question ranged from 4.6 to 5 on the five-point scale plus positive qualitative data from two participants Process variable: Attendance: Of the 14 participants, there were two occasions where participants had to reschedule their phone sessions	Improvement Improvement Satisfied ?	15 – –	High – –
Boardman *et al.* (2014) Single group pre-post-follow-up	SZ	22	✓ Weekly (~20 min/session) ×8 weeks	⨯	Self-reported adherence (missed medication doses previous 4 weeks): Baseline 7.8 (s.d. 5.5, range 5–30), 8-week 1.3 (s.d. 1.4, range 0–4), 14-week 1.2 (s.d. 2.5, range 0–11), *p* = 0.0001	Improvement	Psychiatric symptoms (BPRS). Baseline mean (s.d.) 36 (8.8), 8-week 32 (s.d. 7.7), 14-week 32.2 (6.8), *p* = 0.003 Process variable: Attendance: 22/28 (78.6%) described as completing the intervention	Improvement ?	11	High
Smoking/healthy lifestyles										
Baker *et al.* (2014) Single group pre-post	SZ Spectrum (including BP)	17	✓ 1× (~60 min) + 7× (~26 min) delivered once to twice weekly	⨯	Fruit intake (ARFS): Sig. increase from 5.1 (s.d. = 3.1) to 6.6 (s.d. = 2.9), ES −0.73 Vegetable intake (ARFS): Trend for improvement 12.2 (s.d. = 4) to 13.5 (s.d. = 3.5), ES −0.64 Leisure screen time (weekday time watching television and/or using a computer at home): Sig. reduction from 298 (s.d. = 200) to 163 (s.d. = 107) min/day, ES 0.76	Improvement ? Improvement Improvement	Depression (BDI-FS): 4.5 (s.d. = 3.3) to 3.7 (s.d. = 2.8), ES 0.37, ns Diet quality (ARFS): Sig. increase, 33.2 (s.d. = 10.5) to 38.2 (s.d. = 8.1), ES −0.97 Total servings of fruit and vegetables/day: Increased 4.2 (s.d. = 2.0) to 5.0 (s.d. = 1.8), ES −0.4, ns Time spent walking (IPAQ): 252 (s.d. = 353) to 356 (s.d. = 470) min/day, ES −0.42, ns Overall sitting time: Sig. decrease 555 (s.d. = 191) to 412 (s.d. = 211) min/day, ES 0.73 Smoking: Of five smokers, two reported abstinence at post-treatment. A third reported a 50% reduction in cigarettes per day (ES 1.03 for CPD) Alcohol consumption (2-week TLFB): 1 hazardous drinker at baseline, alcohol use not targeted Cannabis (OTI): three users at baseline, two abstinent post-treatment, one reduced use by 67% (ES 1.02) Quality of life (WHO-8 EUROHIS): 25.6 (s.d. = 5.6) to 28.4 (s.d. = 6.6), ES −0.65, ns Functioning (GAF): Significant improvement 57.1 (s.d. = 6.7) to 62.7 (s.d. = 8.9), ES-0.65 Satisfaction: All participants rated the quality of the service as ‘good’ (17.6%) or ‘excellent’ (82.4%). The majority indicated they were ‘mostly’ (17.6%) or ‘very’ satisfied (76.5%) with one person (5.9%) ‘indifferent or mildly dissatisfied’ Process Variable: Attendance: 19 (95%) completed all eight intervention sessions, with one person withdrawing due to lack of privacy in their boarding house	NSD Improvement ? Improvement ? Improvement Improvement ? Improvement ? ? Improvement NSD Improvement Satisfied ?	14 – – – – – – – – – – –	High – – –– – – – – – – – –

ASRM, Altman Self-Rating Mania Scale; ARFS, Australian Recommended Food Score; BDI-FS, Beck Depression Inventory Fast Screen; BMMQ, Bipolar Mood Management Questionnaire; BP, bipolar; BPRS, Brief Psychiatric Rating Scale; CPD, cigarettes per day; ES, effect size; GAF, Global Assessment of Functioning; IPAQ, International Physical Activity Questionnaire; M, Mean; MARS, Medication Adherence Report Scale; NSD, no significant difference ; OTI, Opiate Treatment Index; QIDS, Quick Inventory of Depressive Symptomatology (-C, clinician rated; -SR, self-rated); s.d., standard deviation; SEAMS, Self-Efficacy for Appropriate Medication Use Scale; SZ, schizophrenia; TCAS, Therapist Competence/Adherence Scale; TLFB, Timeline Follow Back; WHO-8 EUROHIS, Shortened version of the World Health Organisation Quality of Life Instrument-Abbreviated Version.

### Outcomes assessed

Outcome measures utilised in each study are reported in Tables 1 and 2. There was considerable heterogeneity. In studies of relapse prevention, the primary outcome was typically relapse, which was variously defined according to number of days until psychiatric hospitalisation, number of days until DSM criteria (IV or IV-TR) were met for a mood episode [(hypo)mania, depression, mixed)] and/or severity of symptoms. All 10 studies included one or more measures of psychiatric symptomatology, but only three included measures of quality of life and/or functioning (Castle *et al.*, 2007; Javadpour *et al.*, 2013; Wenze *et al.*, 2015) and only one utilised an index of personally meaningful recovery as a primary outcome (Haddock *et al.*, 2017). In studies of medication adherence, the primary outcome was typically medication compliance, as per self-report or clinician administered assessment. Studies typically included one or more measures to assess the impact on symptoms, service utilisation and attitudes (including self-efficacy and insight), but only two assessed the impact on quality of life and/or functioning (Salzer *et al.*, 2004; Montes *et al.*, 2010). In studies of CVD/health risk behaviours, primary outcomes typically included an index of smoking (Baker *et al.*, 2015; Heffner *et al.*, 2015) or CVD risk (Kilbourne *et al.*, 2012; Baker *et al.*, 2015). One study (Baker *et al.*, 2014) focused on sedentary activity and intake of fruit and vegetables. Functioning and/or quality of life were assessed in three of the four studies (Kilbourne *et al.*, 2012; Baker *et al.*, 2014; Baker *et al.*, 2015).

### Methodological quality and risk of bias in included studies

Studies are presented in descending order of methodological quality in Table 1 for controlled trials and Table 2 for single-arm studies. No clear pattern emerged between methodological rigour and whether or not the outcomes were in favour of the telephone condition. Across all trials, there was considerable variation in methodological quality scores on the Downs and Black scale, (total scores ranged from 9 to 25 out of 27). At least half of included studies scored 0 for the following items: adverse events; characteristics of those lost to follow-up; representativeness of the sample; attempts to have blinded outcomes assessors and adequate power (six studies reported power calculations, one had sufficient power). For the 12 RCTs Pedro scores ranged from two to eight out of eight. At least half of included studies scored 0 for ‘blinding of outcomes assessors’, and ‘measures of at least one key outcome variable from at least 85% of original participants’.

Cochrane risk of bias assessments is presented in online Supplementary Fig. S1a and S1b, with overall risk of bias scores in Tables 1 and 2. To summarise, eight studies reported adequate random sequence generation, four reported allocation concealment procedures, four stated that assessors were blinded to intervention status, nine were unlikely to be subjected to attrition bias, and 11 may have been affected by reporting bias. Regarding the overall risk of bias, all non-RCTs were automatically rated as ‘high’ for overall risk of bias. Eight RCTs were rated as having a high overall risk of bias (Beebe, 2001; Salzer *et al.*, 2004; Castle *et al.*, 2007; Price, 2007; Kilbourne *et al.*, 2012; Javadpour *et al.*, 2013; Wenze *et al.*, 2015; Beebe *et al.*, 2016), with all rated as unclear regarding one or both of two key items (sequence generation and allocation concealment). The remaining five RCTs were rated as having a low overall risk of bias, although only two (Simon *et al.*, 2006; Baker *et al.*, 2015) had adequately blinded outcomes assessors and a pre-published protocol.

### Synthesis of results

Results of individual studies are presented in Table 1 (controlled trials) and 2 (single arm studies). Heterogeneity of form of intervention delivery (telephone only or in combination), control group (active or inactive control) and outcome measures precluded a meta-analysis on (within outcomes or collapsed across groups). A narrative synthesis is presented below.

### Effects of Interventions

#### Relapse prevention

Of the 10 trials assessing relapse prevention, there were eight RCTs (Beebe, 2001; Simon *et al.*, 2006; Castle *et al.*, 2007; Price, 2007; Castle *et al.*, 2010; Javadpour *et al.*, 2013; Komatsu *et al.*, 2013; Wenze *et al.*, 2015), one partially randomised preference trial (Haddock *et al.*, 2017) and one open trial (Miklowitz *et al.*, 2012). Numbers in the RCT component of the preference trial were low (only three participants chose to be randomised), therefore this study has been categorised as an observational for the purpose of this review. Five RCTs reported at least 50% of outcomes significantly in favour of the telephone intervention, over time periods of up to 18 months (Javadpour *et al.*, 2013); four relative to an active comparison condition (Castle *et al.*, 2007; Castle *et al.*, 2010; Komatsu *et al.*, 2013; Wenze *et al.*, 2015) and one relative to TAU (Javadpour *et al.*, 2013). For the remaining RCTs, Beebe and colleagues (the only study in which the telephone was the sole delivery method) did not detect significant differences between active treatment conditions on the three indicators of relapse used (Beebe, 2001); Simon and colleagues demonstrated significant effects in favour of the telephone condition in two of the eight outcomes, but otherwise equivalent performance to TAU (Simon *et al.*, 2002; 2005, ) while Price found that the difference seen in hospital admissions and treatment compliance for the telephone condition (relative to TAU) did not reach statistical significance (Price, 2007). For the two non-RCTs, Haddock *et al.*, did not detect significant differences between active treatment conditions and/or TAU for eight of the nine outcomes assessed, with the remaining outcome (Recovery from Negative Impacts of Psychosis) in favour of TAU [although the authors urge caution when interpreting this finding due to multiple comparisons (Haddock *et al.*, 2017)] and Miklowitz *et al*. found significant improvement in knowledge of mood management strategies, but was unable to calculate the statistical significance of observed improvements in mania and depression (Miklowitz *et al.*, 2012).

As seen in Table 1, seven RCTs reported readmission or rehospitalisation data [all except (Castle *et al.*, 2010)], with six in favour of the telephone intervention and three attaining statistical significance (Castle *et al.*, 2007; Javadpour *et al.*, 2013; Komatsu *et al.*, 2013). Six RCTs reported symptom outcomes (Simon *et al.*, 2002; Simon *et al.*, 2005; Simon *et al.*, 2006; Castle *et al.*, 2007; Castle *et al.*, 2010; Javadpour *et al.*, 2013; Komatsu *et al.*, 2013; Wenze *et al.*, 2015), five demonstrated significant advantages of the telephone intervention on at least one symptom (Simon *et al.*, 2002; Simon *et al.*, 2005; Simon *et al.*, 2006; Castle *et al.*, 2010; Javadpour *et al.*, 2013; Komatsu *et al.*, 2013; Wenze *et al.*, 2015).

#### Medication adherence

Of the six trials reporting on medication adherence as the primary outcome three were RCTs (Salzer *et al.*, 2004; Montes *et al.*, 2010; Beebe *et al.*, 2016), one non-randomised (Cook *et al.*, 2008) and two single-group pre-post designs (Boardman *et al.*, 2014; McKenzie and Chang, 2015). For the RCTs, the larger study (Montes *et al.*, 2010) was the only to report at least 50% of outcomes in favour of the telephone condition. Although Salzer (Salzer *et al.*, 2004) demonstrated effect sizes in the direction of the telephone for eight of the ten outcomes evaluated (using an intervention delivered entirely over the telephone). However, the two medication adherence outcomes (subjective response to medication and self-reported treatment adherence) did not significantly differ between groups (Salzer *et al.*, 2004). Similarly, in their entirely telephone-delivered intervention Beebe (Beebe *et al.*, 2016) did not detect a between-group difference for medication adherence. Conversely, in their non-randomised trial of an intervention delivered entirely by telephone, Cook reported improved adherence (both pharmacy based and self-report measures) in favour of the telephone condition (Cook *et al.*, 2008). Both open trials reported improved self-reported medication adherence post-treatment (Boardman *et al.*, 2014; McKenzie and Chang, 2015).

#### Smoking or CVD risk behaviours

There were four studies reporting smoking or CVD risk behaviour outcomes (Kilbourne *et al.*, 2012; Baker *et al.*, 2014; Baker *et al.*, 2015; Heffner *et al.*, 2015), with two RCTs (Kilbourne *et al.*, 2012; Baker *et al.*, 2015) – one of those a pilot trial (Kilbourne *et al.*, 2012). Both RCTs utilised an active comparison condition and neither demonstrated at least 50% of outcomes in favour of the telephone condition. Baker *et al.* (2015), demonstrated significant improvements in CVD risk and smoking at 12 months following either a largely telephone-delivered intervention or a multi-component face-to-face intervention. Significant improvements in global functioning were also seen in both conditions (Baker *et al.*, 2015). Neither condition demonstrated significant improvements in health behaviours other than smoking (Baker *et al.*, 2015). Cardiometabolic risk (BMI and blood pressure) and health-related quality of life also remained stable for both conditions in the pilot RCT by Kilbourne *et al.* (2012) and between-group differences for functioning and depression symptoms approached significance, in favour of the telephone condition (Kilbourne *et al.*, 2012). Further, for individuals at greater risk (BMI⩾30 or systolic BP>140), *post hoc* analyses demonstrated superior improvement in functioning and depressive symptoms for the telephone condition (Kilbourne *et al.*, 2012). For the single-arm studies, results from Heffner *et al.* (2015) suggest largely equivalent performance of the phone and face-to-face delivery for a smoking cessation intervention, although between groups comparisons were not performed. Finally, in a single-group pre-post design Baker *et al.* (2014) demonstrated clinically important change across a range of health behaviours following an intervention delivered entirely by telephone.

## Discussion

This review aimed to capture all relevant studies of interventions delivered on at least 50% of session occasions by telephone to improve relapse prevention, medication adherence or reduce smoking and/or other CVD risk behaviour. We sought to comment on the feasibility and efficacy of telephone-delivered psychosocial interventions in people with a psychotic disorder. A total of 20 trials were reviewed in full, with 13 RCTs. Overall, the literature is split relatively evenly across schizophrenia or schizoaffective disorder and bipolar disorder. Studies typically included one or more ‘traditional’ clinical outcomes (e.g. symptomatology, relapse, medication compliance), with considerably fewer assessing the quality of life or functioning. Little is known about the process variables that may influence treatment outcome and only one study conducted economic analysis.

Although the modest body of literature and diversity of methods precludes definitive comments on efficacy, positive effects were found. Five of eight RCTs evaluating relapse prevention and one of three RCTs evaluating medication adherence reported at least 50% of outcomes in favour of the telephone-delivered the intervention, for time periods up to 18 months. As for smoking and other CVD risk behaviour studies, comparable levels of improvement were seen across treatment conditions. Of note, the comparison condition for one of the studies (Baker *et al.*, 2015) was an intensive, multi-component face-to-face delivered intervention with longer session duration. Accordingly, the equivalent level of improvement seen is important and points to the potential efficiency of telephone-delivered interventions for promoting clinically meaningful change.

The results in each domain of relapse prevention, medication adherence and smoking and CVD risk behaviour interventions are encouraging. Although most interventions combined telephone and face-to-face delivery, there were indications that entirely telephone-delivered interventions might be effective (e.g. Baker *et al.*, 2014, Boardman *et al.*, 2014), with evidence of at least equivalent (Beebe, 2001; Beebe *et al.*, 2016) if not superior performance (Salzer *et al.*, 2004; Cook *et al.*, 2008) relative to standard care. In addition, in the relapse prevention preference trial conducted by Haddock *et al.*, (2017), strong preferences were nominated by study participants for either telephone or telephone plus group delivery, with a significantly greater number of telephone sessions attended in the telephone only condition and few group sessions attended, on average. Thus, this review suggests that telephone-delivered interventions may be popular among service users, well attended, and at least as effective, if not superior to treatment as usual. Clearly, further methodologically rigorous research is warranted.

### Limitations

Firstly, this review identified a modest sample of heterogeneous studies. Differences in outcome assessment, intervention and comparator conditions precluded meta-analysis. Accordingly, it is difficult to draw strong conclusions about the impact of telephone-delivered interventions on the outcomes of interest. There was also considerable variation in methodological quality. Most studies were uncontrolled and less than half of the RCTs identified were deemed to be at low risk of bias. In addition to poor reporting around randomisation and allocation concealment, many studies did not report using blinded outcomes assessors. Adequately powered RCTs were also rare. Many had small sample sizes, and all but one of those reporting power calculations were underpowered to detect significant differences. The cross-cultural generalisability of our findings is also restricted as we limited our search to English language publications.

### Implications for practice

Despite psychological interventions being recommended (Galletly *et al*., 2016; National Institute for Health and Care Excellence, 2014*a*, 2014*b*) for the treatment of schizophrenia and other psychotic disorders, of those likely to benefit, only 10% or less have access (Gulliver *et al.*, 2010; Haddock *et al.*, 2014; Schizophrenia Commission, 2015). Our findings lend further support to the potential role of phone delivered interventions in improving access. Importantly, the treatment protocols included in the current review were delivered by a variety of health professionals and ranged from brief time-limited ‘check-in's’ (e.g. Price, 2007) to full psychological interventions (e.g. Baker *et al.*, 2014). Accordingly, telephone delivery may help to overcome barriers related to accessibility of support services and availability of trained clinicians (Gulliver *et al.*, 2010; Haddock *et al.*, 2014; Schizophrenia Commission, 2015), while maintaining the verbal contact and social connectedness of face-to-face delivery. Moreover, contrary to reservations from service providers, especially with regards to severe mental illness [SMI (Perle *et al.*, 2013)], evidence from the current, and other (Kasckow *et al.*, 2014) reviews suggest that telephone interventions are acceptable and well attended by adults with SMI.

### Implications for research

To better establish the effectiveness of telephone interventions for people with a psychotic disorder, high quality, adequately powered studies are an important priority. The latter might best be conducted within existing practice settings to better evaluate the real-world impact of telephone-delivered interventions. To better understand the comparative clinical and cost effectiveness of telephone-delivered interventions, more head to head trials are needed. This would also help inform what, if any modifications are needed to ensure that telephone-delivered interventions meet the needs and preferences of service users. With the increasing focus on peer workers in mental health services, future research may also benefit from examining the acceptability and effectiveness of using peer workers to deliver telephone interventions. While it is challenging in studies of psychological interventions to use a double-blind design, the use of blinded outcomes measurement [e.g. a prospective, randomised, open, blinded endpoint (PROBE) design] has been argued to be a sufficient alternative (Hansson *et al.*, 1992). Greater attention to non-symptom indicators of wellbeing (e.g. quality of life and functioning) and process variables (e.g. therapeutic alliance) is also warranted. To allow comparison between studies, greater uniformity in outcome measures would be beneficial. Accordingly, agreement upon and adherence to standard definitions of common outcome variables is an important priority for future research.

## Supplementary material

For supplementary material accompanying this paper visit http://dx.doi.org/10.1017/S0033291718001125. click here to view supplementary material

## Registration

PROSPERO: International prospective register of systematic reviews Registration Number: CRD42015025402 Date: 17 August 2015
