# Supplementary material for: Telephone-delivered psychosocial interventions targeting key health priorities in adults with a psychotic disorder: systematic review
Source: Psychol Med. 2018 May 25;48(16):2637–57. doi: 10.1017/S0033291718001125 (PMC6236444; doi:10.1017/S0033291718001125)
Supplement: Supplementary file 1 [file S0033291718001125sup001.zip › Appendix 1_Medline Search Strategy.docx]

Appendix 1

Medline Search Strategy

| **Date** | **Database** | **Search Strategy** | **Notes** |
| --- | --- | --- | --- |
| 14/05/2015 | MEDLINE | Telephone [MH] OR  ("telephone intervention"[Title] OR "phone intervention"[Title] OR "telephone program"[Title] OR "phone program"[Title] OR "telephone trial"[Title] OR "phone trial"[Title])  OR  ("telephone intervention"[Abstract] OR "phone intervention"[Abstract] OR "telephone program"[ Abstract] OR "phone program"[Abstract] OR "telephone trial"[Abstract] OR "phone trial"[Abstract])  AND  Psychosis[MH] OR schizophrenia [MH] OR psychosis [Title] OR schizo*[ Title] OR bipolar [Title]OR psychosis [Abstract] OR schizo*[Abstract] OR bipolar [Abstract]  AND  Cardiovascular[MH] OR diet [MH] OR nutrition [MH] OR physical activity [MH] OR exercise [MH] OR smoking[MH] OR medication compliance [MH] OR alcoholism[MH] OR alcohol-related disorders[MH] OR substance-related disorder[MH] OR relapse prevention [MH]  OR  Cardiovascular [Title] OR dietary intake [Title] OR diet [Title] OR nutrition [Title] OR fruit [Title] OR physical activity [Title] OR exercise [Title] OR smoking [Title] OR medication compliance [Title] OR alcoholism [Title] OR alcohol abuse [Title] OR alcohol dependence [Title] OR substance abuse [Title] OR substance dependence [Title] OR addiction [Title] OR smok* [Title] ] OR relapse prevention [Title]  OR  Cardiovascular [Abstract] OR dietary intake [Abstract] OR diet [Abstract] OR nutrition [Abstract] OR fruit [Abstract] OR physical activity [Abstract] OR exercise [Abstract] OR smoking [Abstract] OR medication compliance [Abstract] OR alcoholism [Abstract] OR alcohol abuse [Abstract] OR alcohol dependence [Abstract] OR substance abuse [Abstract] OR substance dependence [Abstract] OR addiction [Abstract] OR smok* [Abstract] relapse prevention [Abstract] | Limits:  English Language |
