# Supplementary material for: Telephone-delivered psychosocial interventions targeting key health priorities in adults with a psychotic disorder: systematic review
Source: Psychol Med. 2018 May 25;48(16):2637–57. doi: 10.1017/S0033291718001125 (PMC6236444; doi:10.1017/S0033291718001125)
Supplement: Supplementary file 1 [file S0033291718001125sup001.zip › Supplementary File 1_Process Variables_01.03.2018.docx]

Supplementary File 1

Results

Process Variables

Attendance was the most commonly reported process variable [15 studies (Simon *et al.*, 2002, Salzer *et al.*, 2004, Simon *et al.*, 2005, Simon *et al.*, 2006, Price, 2007, Cook *et al.*, 2008, Montes *et al.*, 2010, Kilbourne *et al.*, 2012, Miklowitz *et al.*, 2012, Javadpour *et al.*, 2013, Baker *et al.*, 2014, Boardman *et al.*, 2014, Baker *et al.*, 2015, Heffner *et al.*, 2015, McKenzie and Chang, 2015, Wenze *et al.*, 2015, Haddock *et al.*, 2017). Four were single arm trials (Miklowitz *et al.*, 2012, Baker *et al.*, 2014, Boardman *et al.*, 2014, McKenzie and Chang, 2015) and eight did not compare between treatment arms – details were typically provided only for the intervention condition (Salzer *et al.*, 2004, Simon *et al.*, 2005, Price, 2007, Cook *et al.*, 2008, Montes *et al.*, 2010, Kilbourne *et al.*, 2012, Javadpour *et al.*, 2013, Wenze *et al.*, 2015)]. Of the three studies that reported between group comparisons, there was one focused on relapse prevention (Haddock *et al.*, 2017) and two on smoking/ CVD risk (Baker *et al.*, 2015, Heffner *et al.*, 2015). Attendance was higher for those in the telephone condition in two studies (Baker *et al.*, 2015, Haddock *et al.*, 2017) and lower in one (Heffner *et al.*, 2015). Only one study explored the relationship between attendance and treatment outcome (Baker *et al.*, 2015). Higher attendance was associated with improved treatment outcomes in CVD risk and smoking behaviour (Baker *et al.*, 2015).

Treatment satisfaction was reported in two of the relapse prevention studies (Wenze *et al.*, 2015, Haddock *et al.*, 2017), two of the medication adherence studies (Salzer *et al.*, 2004, McKenzie and Chang, 2015) and two of the smoking/ CVD studies (Baker *et al.*, 2014, Heffner *et al.*, 2015), but the relationship to treatment outcome was never assessed. Overall satisfaction with the various telephone interventions was high, with two studies finding higher levels of satisfaction in the phone relative to the comparison condition (Heffner *et al.*, 2015, Wenze *et al.*, 2015).

Additional process variables included treatment preference and therapeutic alliance (Haddock *et al.*, 2017), acceptance (Heffner *et al.*, 2015) and motivation to change (McKenzie and Chang, 2015). Regarding treatment preference, Haddock and colleagues found that the proportion of individuals selecting a phone based intervention was roughly equivalent to those wishing to continue treatment as usual (Haddock *et al.*, 2017). Ratings of therapeutic alliance and acceptance were high and did not statistically differ between telephone and comparison conditions (Heffner *et al.*, 2015, Haddock *et al.*, 2017). Motivation to change was only explored in a single, one arm study (McKenzie and Chang, 2015), with significant improvements over time in participant motivation to adhere to their medication regime following a telephone delivered intervention. Although again, the potential impact of these variables on treatment outcome was never assessed.

Only one study (focused on relapse prevention) included an economic analysis (Simon *et al.*, 2006). Simon et al found that their telephone intervention cost approximately $800 to deliver and relative to treatment as usual, was associated with greater costs for outpatient mental health visits and psychotropic prescription but considerable savings on psychiatric hospitalisation (no differences attained statistical significance). The authors concluded that the additional costs were ‘modest’ and could be balanced against observed clinical benefits (additional 5.5 weeks free from significant symptoms of mania).
