# Supplementary material for: Telephone-delivered psychosocial interventions targeting key health priorities in adults with a psychotic disorder: systematic review
Source: Psychol Med. 2018 May 25;48(16):2637–57. doi: 10.1017/S0033291718001125 (PMC6236444; doi:10.1017/S0033291718001125)
Supplement: Supplementary file 1 [file S0033291718001125sup001.zip › Supplementary Table 1_01.03.2018.docx]

Table 1. Summary of Participant Characteristics and Treatment Details as a Function of Study Focus (Relapse Prevention vs. Medication Adherence vs. Smoking/ Healthy Lifestyles) and Comparison Condition (Active vs. Treatment as Usual)

| **Study**  **Setting, Country** | **N**  **(n phone)** | | **Mean Age**  **(years)** | **% Male** | | | | **% White** | | | **Clinical Characteristics** | | | **Intervention Summary** | | | | | **Summary of control Sessions** |  |  |  |  |
| --- | --- | --- | --- | --- | --- | --- | --- | --- | --- | --- | --- | --- | --- | --- | --- | --- | --- | --- | --- | --- | --- | --- | --- |
| **RELAPSE PREVENTION** | | | | | | | | | | | | | | | | | | | | |  |  |  |
| ***Telephone vs Active Comparison Condition*** | | | | | | | | | | | | | | | | | | | | |  |  |  |
| **Beebe (2001)**  Community setting  USA | 37 (15) | | 40.4 | 73.0% | | | | 76.0% | | | DSM-IV diagnosis of schizophrenia; no details on how diagnosis was made.  Mean of 5.8 prior psychiatric hospital admissions  Post discharge from psychiatric hospital. | | | Delivered alongside routine community-based follow-up care focussing on: attendance at appointments, medication, symptoms; new issues/worries/ any other topics.  **Session frequency/length:**  weekly, about 10 mins/session.  **Duration:** 3 months (commencing about one month post-discharge).  **Delivered by:** nurse investigator. | | | | | Routine community-based follow-up care & informational phone contact (1-3 mins) at 6 & 12 weeks from nurse investigator. |  |  |  |  |
| **Castle et al (2007)**  Outpatient setting  Australia | 17 (8) | | 44.0 | 17.6% | | | | Not reported | | | Diagnosis of Bipolar I (58.8%) or II (41.2%) on the MINI.  Under the management of a medical practitioner  Receiving usual pharmacological treatment. | | | Group sessions (manual guided) & telephone checks (prompt attendance & assist with homework). Groups comprised education, peer support, new coping strategies & relapse prevention.  **Session frequency/length**: weekly; group 90 mins/session, telephone not reported; 3 group booster sessions conducted at monthly intervals.  **Duration:** 12 weeks (excl. booster sessions).  **Delivered by:** research assistants with training & clinical experience in group delivery. | | | | | Weekly phone calls (content not specified) controlling for extra contact time with researchers outside of the structured group intervention. |  |  |  |  |
| **Castle et al (2010)**  Outpatient  Setting  Australia | 82 (32) | | 42.1 | 23.8% | | | | Not reported | | | DSM-IV-TR criteria for Bipolar I (73.8%) or II (25%), or not otherwise specified (1.2%) on the MINI.  Average age of diagnosis 32 years, (10 years prior to the study)  Number of weeks spent in hospital in previous 12 months: ~2 weeks. | | | Intervention description, session frequency, length & duration as above (Castle et al 2007).  **Delivered by:** one senior research clinician from the project team & a clinician recruited from a clinical site. | | | | | As above |  |  |  |  |
| **Komatsu et al (2013)**  Outpatient setting  Japan | 45 (22) | | 43.1 | 55.5% | | | | Not reported | | | DSM-IV-TR diagnosis of schizophrenia (diagnostic interview not specified, likely clinical diagnosis)  Outpatients with a mean period following last hospital discharge of 35 months (SD 61, telephone) and 46 months (SD 51, control) | | | 10-item Early Warning Sign Questionnaire (EWSQ) via weekly phone calls. If the computer-generated score exceeded a given threshold, patients were instructed to increase their antipsychotic by 20% of baseline within the next 24 hours. Nurses visited patients' homes to verify increases in oral medication.  **Session frequency:** Weekly.  **Duration:** 12 months  **Delivered by:** Nurses | | | | | Weekly phone calls to administer the EWSQ. Nursing home visits conducted as usual (focusing on symptoms & recommending early medical examinations) whether or not the EWSQ predicted relapse. |  |  |  |  |
| **Wenze et al (2015)**  Outpatient setting  USA | 30 (14) | | 46.9 | 50% | | | | 89.75% | | | DSM IV diagnosis of Bipolar I or II Disorder or Bipolar Disorder NOS as determined by the SCID.  Baseline mood state (depressed) 6 (42.9%) telephone vs. 12 (75%) control  Outpatients post-discharge from psychiatric hospital | | | Manual guided. Individual in-person sessions focused on treatment & psychiatric history, psycho-education, personal values, goals & life plan. An in-person family meeting focused on psychoeducation & supporting treatment. Telephone contact reviewed symptoms, substance use, treatment, challenges & problem-solving. Community Treatment providers received regular feedback letters.  **Session frequency/length:** Telephone: weekly for the first month & then at decreasing frequency, 15-30 minutes/ session. Individual in-person: 3 x 1 hour sessions. Family: 1 hour.  **Duration:** 6 months; 11 telephone sessions.  **Delivered by:** doctoral level clinicians, trained until judged competent. | | | | | Enhanced assessment & monitoring - a battery of assessments were administered as part of the study procedures & treatment providers given a written summary. |  |  |  |  |
| ***Telephone vs Treatment as Usual*** | | | | | | | | | | | | | | | | | | | | |  |  |  |
| **Haddock et al (2017)**  Outpatient setting  UK | 89 (68) | | 36.0 | | 63% | | | 80.5% | | | ICD-10 diagnosis of schizophrenia spectrum disorder (diagnostic interview not specified). Schizophrenia 41%, schizoaffective disorder 13%, psychosis not otherwise specified 36%, paranoid schizophrenia 6%, and bipolar disorder 1%.  Symptoms stable for one month | | | Telephone Supported Therapy (TS): TAU + manual guided primarily telephone delivered recovery oriented CBT incorporating a self-help manual.  High Support (HS): TAU + TS + manual guided group sessions that focused on shared ideas & experiences & explored recovery, CBT principles & normalising experiences.  **TS Delivered by:** CBT Therapist  **TS Session frequency/length:**  Weekly, up to 1 hour.  **HS Delivered By:** CBT therapist & a researcher with lived experience of mental health challenges.  **HS Session frequency/length:**  As per TS + up to 12 bi-weekly, 2-hour group sessions delivered over 6 months | | | | | TAU: usual medical & nursing care management plus trial assessments at baseline, 9- & 15-months |  |  |  |  |
| **Javadpour et al (2013)**  Outpatient setting,  Iran | 108 (54) | | Not reported | | 49% | | | Not reported | | | Hospital discharge diagnosis of bipolar disorder.  Participants were in the remission phase following discharge from hospital. | | | Standard pharmacotherapy for bipolar disorder plus individual face-to-face psycho-education on bipolar disorder, relapse prevention signs and strategies, plus monthly telephone contact to remind the participant of their next appointment & respond to queries.  **Session frequency/length:** Face-to-face: 8 weekly sessions, 50 mins/session; Telephone: monthly, 10 mins/session.  **Duration:** 18 months.  **Delivered by:** The same psychiatry resident in the psychology clinic at a teaching hospital. | | | | | TAU: Standard pharmacotherapy of their psychiatrists' choice for 18 months. |  |  |  |  |
| **Price (2007)**  Transition from inpatient to community setting  USA | 13 (7) | | 25.5 | | 69% | | | 69% | | | Participants had a diagnosis of schizophrenia or schizoaffective disorder consistent with DSM-IV. | | | Part 1: Structured interview 2-3 days before discharge to determine plans for housing, employment or education; assess knowledge about the outpatient site for follow-up appointments; discuss prescribed medications; discuss any concerns with medication or follow-up appointments. Part 2: Phone calls to check on welfare & to remind about clinic appointment. Part 3: Participants provided with a prepaid mobile phone and encouraged to call the nurse if any problems arose. Part 4: Nurse called the case manager to review information from Part 1.  **Session frequency:** Telephone: twice between discharge & scheduled outpatient appointment; nurse available 24 hours/day during first 2 weeks & as needed during following 4-6 weeks.  **Delivered by:** Advanced practice psychiatric nurse. | | | | | TAU: Structured interview with no further nurse-client contact. |  |  |  |  |
| **Simon et al (2006)**  Mental health clinics,  USA | 441 (212) | | 44.2 | | 31.7% | | | 88.4% | | | Diagnosis of Type I or Type II Bipolar Disorder by DSM-IV criteria using the SCID interview, or by clinical record review if SCID did not confirm diagnosis.  98 participants (22.2%) were in remission (mania and depression PSR scores, 1-2),  183 participants (41.5%) met threshold for current major depression, hypomania, or mania  160 participants (36.3%) had subthreshold mood symptoms | | | In-person visit to the nurse care manager for development of a collaborative structured care plan including warning signs & coping strategies + Telephone monitoring: clinical & self-reported mood ratings; medication use/ side-effects; follow-up plans; brief support & review of problems discussed at group meetings + Provision of written, structured feedback & medication recommendations to treatment providers + outreach & support as needed + Group program (psychoeducation & problem solving).  **Session frequency/duration:** Telephone: once/month (additional ‘as needed’); Group: bi-monthly, 1 hour; outreach visits as needed.  **Duration:** up to 24 months  **Delivered by:** 3 nurse care managers delivered the intervention & collaborated with regular care providers. | | | | | TAU: existing treatment & any or all services normally available at 2/4 participating clinics, unstructured support groups for patients with bipolar disorder were available |  |  |  |  |
| **Miklowitz et al (2012)**  Outpatient setting,  UK | 19 (19) | | 37.2 | | 31.6%; | | | 94.7% | | | Patients were referred to the program with DSM-IV-TR diagnoses of bipolar I (73.7%) or II (26.3%). Diagnostic interview not specified, likely clinical diagnosis. | | | Manual guided psychoeducation (identifying relapse signature; reviewing risk & protective factors; daily rhythm & sleep/wake regulation; role of medications & substance/alcohol use; finalizing the mood management plan). Participants were encouraged to invite a caregiver to sessions. Telephone calls focused on checking in & whether they had been implementing the plan. Daily texts & emails to monitor mood & sleep, and weekly ratings of depression & mania  **Session frequency/length:** Pilot I: 6 weekly sessions. Pilot II: 5 weekly sessions. Bi-weekly & then monthly 30min phone calls for 3 months after the final session. Daily texts/emails.  **Duration:** 4-5 months.  **Delivered by:**  University qualified facilitators with no formal clinical qualifications, trained using a three-day workshop & bi-weekly group supervision. | | | | | N/A |  |  |  |  |
| **MEDICATION ADHERENCE** | | | | | | | | | | | | | | | | | | | | |  |  |  |
| ***Telephone vs Active Comparison Condition*** | | | | | | | | | | | | | | | | | | | | |  |  |  |
| **No Studies** | | | | | | | | | | | | | | | | | | | | |  |  |  |
| ***Telephone vs. Treatment as Usual*** | | | | | | | | | | | | | | | | | | | | |  |  |  |
| **Beebe et al (2016)**  Community health centre,  USA | | 140 (?) | 46.1 | | | 57.1% | | | 62.1% | | | Schizoaffective 67.1%, schizophrenia 32.9% chart diagnosis according to DSM-IV. Diagnostic interview not specified.  Not hospitalised for psychiatric illness within the past 6 months. | | | The theory of planned behaviour guided intervention. Delivered by nurses who expressed & reinforced the value of adherence, educated the participant about adherence benefits, & problem solved adherence barriers.  **Session frequency:** Once/week.  **Duration:** 3 months.  **Delivered by:** Nurses. | | Usual care with no telephone intervention. | | | |  |  |  |
| **Cook et al (2008)**  Managed Medicaid health plan for women and children  Mid-Western state - USA | | 202 (51) | 33.4 | | | 16.2% | | | Not reported | | | Self-reported diagnoses: bipolar disorder (22/58; 37.9%), depression (18/58; 31.0%), anxiety disorders (10/58; 17.2%), schizophrenia (2/58; 3.4%), schizoaffective disorder 1/58; 1.7%), hallucinations and delusions (3/58; 5.2%), antisocial personality (1/58; 1.7%), and borderline personality (1/58; 1/7%), 1 missing. | | | Initial calls to screen for non-adherence risk. Low risk participants received a toll-free phone number plus 1 follow-up call at 6 months. High-risk participants received follow-up calls. Strategies included MI or CBT counselling based on readiness to change & individual barriers to adherence. Written materials mailed to 88%. A written progress note was sent to their health plan case manager after each call.  **Session frequency/length:** see *Attendance*.  **Duration:** 6 months.  **Delivered by:** Registered nurses. | | No intervention. The control condition consisted of health plan members who could not be contacted with the aim of ruling out regression to the mean, history, and maturation effects. | | | |  |  |  |
| **Montes et al (2010)**  Community mental health centres  Spain | | 928 (456) | 40.1 | | | 65.3% | | | Not reported | | | DSM-IV-TR diagnosis of schizophrenia as established by the SCID.  Mean years from last hospitalization was 3.2 (SD 4.3) telephone and 3.2 (SD 3.7) control.  Average duration of illness was 13.5 years (SD 9.7) telephone and 12.7 years (SD 9.4) control.  Clinically stable (i.e. no changes in severity or new treatments initiated in the last 6 months). | | | Brief semi-structured assessment of treatment [knowledge of medication regimen & adherence] plus the DAI-10. The psychiatrist received a structured report of each contact. Any documented non-adherence prompted a scheduled psychiatrist visit within 7 days.  **Session frequency:** 4, 8 & 12 weeks from baseline. **Delivered by:** Experienced outpatient mental health nurses who knew the patient (training comprised 40 minutes’ didactic instruction from investigators). | | 4-month psychiatrist visit without the telephone calls. | | | |  |  |  |
| **Salzer et al (2004)**  Community Mental Health Centres  USA | | 32 (18) | Not reported | | | Not reported | | | Not reported | | | DSM-IV diagnosis of schizophrenia spectrum disorder. | | | Phone only intervention. Goals included verbal reinforcement for positive self-care behaviours (e.g., adherence), encouraging discussion, validating treatment experience, and problem-solving improvements in self-care. Aimed to improve communication, insight into illness, attitudes & knowledge about treatment, treatment satisfaction, & decrease side effects.  **Session frequency/length:** weekly, <=10 mins/session). **Duration:** 52 weeks. **Delivered by:** Not reported. | | Not described but likely to be TAU | | | |  |  |  |
| ***Telephone With no Comparison Condition*** | | | | | | | | | | | | | | | | | | | | |  |  |  |
| **Boardman et al (2013)**  Community Mental Health Centre  Australia | 28 (28) | | 35.1 | | | 67.9% | | | Not reported | | | Primary diagnosis of schizophrenia from a CMHC.  Baseline mean total BPRS score (administered by their case manager) = 36.0, indicating very mild symptoms of mental illness. | | | Peer delivered problem solving & mutual support approach to address problems with medication adherence.  **Session frequency/length:** weekly, approx. 20 minutes/session.  **Duration:** 8 weeks.  **Delivered by:** Peers (peers had max. two consumers at any given time) trained using an interactive 3-hour work shop & weekly telephone support | | NA | | | |  |  |  |
| **McKenzie & Chang (2014)**  Private outpatient psychiatry office  New York, USA | 14 (14) | | 35.0 | | | 26.7% | | | 100% | | | Diagnosis of bipolar disorder assumed based on the clinical knowledge of the psychiatric provider.  Private psychiatry outpatients with a medication adherence rate of 80% or less over the previous month.  Acute psychosis and suicidal ideation excluded. | | | A 3-week MI intervention. One face-to-face session then two telephone sessions following a script. Participants were encouraged to consider risks & benefits of medication adherence, readiness to change medication taking behaviours, and to review goals.  **Sessions frequency/length:** 1 face-to-face (45-60 minutes) & 2 telephone sessions (20-30 minutes). **Duration:** 3 weeks.  **Delivered by:** nurses | | NA | | | |  |  |  |
| **SMOKING/ HEALTHY LIFESTYLES** | | | | | | | | | | | | | | | | | | | | |  |  |  |
| ***Telephone vs Active Comparison Condition*** | | | | | | | | | | | | | | | | | | | | |  |  |  |
| **Baker et al (2015)**  Community setting  Australia | 235 (113) | | 41.6 | | | | 59% | | | Not reported | | | Diagnosis of schizophrenia spectrum (59%) or bipolar disorder (22%), or nonorganic psychotic syndrome (19%) as confirmed by the MINI.  Average duration of psychosis was 18.6 years (SD 11.6).  Outpatients with a stable psychotic disorder, taking antipsychotic medication as prescribed for at least 2 months, with intention to continue for the duration of the study | | | Manual guided. Initial face-to-face session: MI, feedback (smoking & other CVD risk factors) & case formulation regarding CVD status & unhealthy behaviours. Phone calls monitored anti-psychotic medication side-effects, nicotine withdrawal, distress, smoking behaviour, diet & physical activity.  Week 4 & 8 were face-to-face (to provide NRT). NRT was available for up to 24 weeks  **Session frequency/ length:** Initial face to face session 90 minutes; then 7 x weekly, 3 x fortnightly, 6 x monthly scheduled to be around 10 minutes each. **Duration:** 24 weeks.  **Delivered by:** Psychologists | | Manual guided. “Healthy Lifestyles” (HL) initial in-person session: MI, feedback & case formulation regarding CVD status and risk behaviours. In-person MI & CBT (physical activity, smoking, & healthy eating habits). Contingent reinforcement for CO reduction & abstinence. NRT was available for up to 24 weeks.  **Session frequency/ length:** Initial face to face session 90 minutes; then 7 weekly, 3 fortnightly, 6 monthly scheduled to be around 1 hour each.  **Duration:** 24 weeks.  **Delivered by:** Psychologists | | | |  |  |
| **Heffner et al (2015)**  Community setting  USA | 16 (6) | | 46.5 | | | | 18.5% | | | 95% | | | Bipolar 1 disorder: 67% (telephone), 30% (in-person). Remainder Bipolar II. Diagnosis was made with the MINI (telephone) or SSAGA (in-person).  Had not been admitted to a psychiatric hospital for at least 3 months at the time of screening. | | | Manual guided. ACT core processes of acceptance (willingness to engage in all aspects of experience) & commitment (behaviour driven by personal values). Session activities included mindfulness, cognitive defusion, & identification of personal values. Both in-person & telephone conditions provided with a standard 8-week course of NRT patches (4 weeks at 21mg, 2 weeks at 14mg, 2 weeks at 7mg) starting at session 3 of the protocol.  **Session frequency/ length:** weekly, 30 minutes. **Duration:** 10 sessions.  **Delivered by:** Chief investigator & masters-level counsellor. | | As per face-to face intervention | | | |  |  |
| **Kilbourne et al (2012)**  Community setting  USA | 68 (34) | | 45.3 | | | | 39% | | | 78% | | | Clinical diagnosis of bipolar I, II or not otherwise specified.  Mean score of 18.8 (SD 8.7) on the WHO Disability Assessment Scale. | | | Manual guided. Group self-management sessions featured guided discussions & exercises designed to help participants set self-management goals. MI & CBT techniques were employed to help effectively manage bipolar disorder & cardiometabolic risk factors. Individual telephone or in-person contact tracked symptoms & progress towards wellness goals. Care managers were alerted of healthcare needs & goals.  **Session frequency/length:** Group sessions – 4 x 2 hour weekly then telephone – monthly, 20 mins.  **Duration:** 6 months.  **Delivered by:** The interventionist held a master's degree in social work. | | Enhanced treatment as usual (monthly mailings on wellness topics) + available mental health care & referral to off-site primary care services. | | | |  |  |
| ***Telephone vs. Treatment as Usual*** | | | | | | | | | | | | | | | | | | | | | | | |
| **No studies** | | | | | | | | | | | | | | | | | | | | | | | |
| ***Telephone With no Comparison Condition*** | | | | | | | | | | | | | | | | | | | | | | | |
| **Baker et al (2014)**  Community setting  Australia | 20 (20) | | 33.9 | | | 53% | | | Not reported | | | DSM-IV diagnoses using the DIP; 65% schizophrenia; 18% schizoaffective disorder, bipolar type; 6% major depressive episode with psychosis; 6%psychotic disorder NOS.  Mean time since illness onset (not including prodromal phase) was 12.4 years (SD 5.2, range 3-21).  Outpatients with current mental health treatment and being prescribed psychotropic medication. | | | Manual guided. Initial session utilised MI to provide feedback (fruit & vegetable intake, sedentary time, smoking & alcohol use) & identify goals. Sessions 2-8 consisted of MI & behavioural strategies (behavioural activation, self-monitoring), and check in of mental health symptoms & any side effects. Contingency management for session completion. Resources booklet (goal record forms, lifestyle diary & CVD risk information).  **Session frequency/length:** 1-2/week; session 1 was an average of 60 minutes (SD 15) & the remaining 7 sessions were an average of 26 minutes (SD 9). **Duration**: 8 sessions  **Delivered by:** psychologists. | | NA | | | | | |  |

Note. MI = Motivational Interviewing; CBT = Cognitive Behaviour Therapy; ACT = Acceptance and Commitment Therapy; NRT = Nicotine Replacement Therapy; CVD = Cardiovascular Disease; CO = Carbon Monoxide; DNA = Did Not Attend
