# Supplementary figures and images for: Telephone-delivered psychosocial interventions targeting key health priorities in adults with a psychotic disorder: systematic review
Source: Psychol Med. 2018 May 25;48(16):2637–57. doi: 10.1017/S0033291718001125 (PMC6236444; doi:10.1017/S0033291718001125)

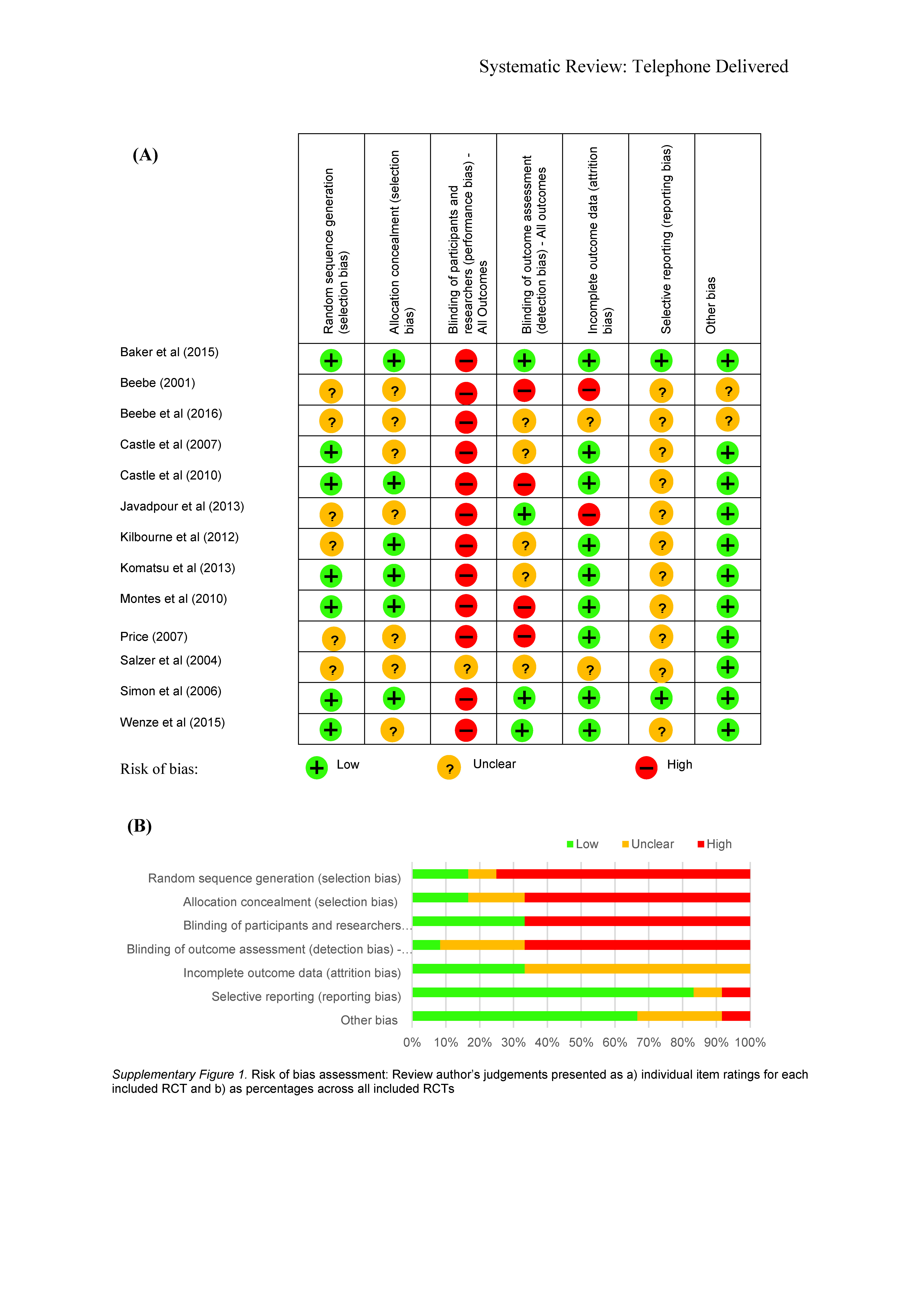

Supplement: Supplementary file 1 [file S0033291718001125sup001.zip › Supplementary Figure 1_RiskofBias_01.03.jpg]
